# Biomechanical models in the lower-limb exoskeletons development: a review

**DOI:** 10.1186/s12984-025-01556-5

**Published:** 2025-01-24

**Authors:** Vahid Firouzi, Andre Seyfarth, Seungmoon Song, Oskar von Stryk, Maziar Ahmad Sharbafi

**Affiliations:** 1 Department of Computer Science, TU Darmstadt, Darmstadt , Germany; 2 Institute of Sport Science, TU Darmstadt, Darmstadt , Germany; 3 Department of Mechanical and Industrial Engineering, Northeastern University, Boston, MA USA

**Keywords:** Assistive device, Biomechanical model, Exoskeleton, Lower-limb, Neuromuscular model

## Abstract

Lower limb exoskeletons serve multiple purposes, like supporting and augmenting movement. Biomechanical models are practical tools to understand human movement, and motor control. This paper provides an overview of these models and a comprehensive review of the current applications of them in assistive device development. It also critically analyzes the existing literature to identify research gaps and suggest future directions. Biomechanical models can be broadly classified as conceptual and detailed models and can be used for the design, control, and assessment of exoskeletons. Also, these models can estimate unmeasurable or hard-to-measure variables, which is also useful within the aforementioned applications. We identified the validation of simulation studies and the enhancement of the accuracy and fidelity of biomechanical models as key future research areas for advancing the development of assistive devices. Additionally, we suggest using exoskeletons as a tool to validate and refine these models. We also emphasize the exploration of model-based design and control approaches for exoskeletons targeting pathological gait, and utilizing biomechanical models for diverse design objectives of exoskeletons. In addition, increasing the availability of open source resources accelerates the advancement of the exoskeleton and biomechanical models. Although biomechanical models are widely applied to improve movement assistance and rehabilitation, their full potential in developing human-compatible exoskeletons remains underexplored and requires further investigation. This review aims to reveal existing needs and cranks new perspectives for developing more effective exoskeletons based on biomechanical models.

## Introduction

Lower-limb exoskeletons offer a variety of applications. They could significantly improve mobility and the quality of life for individuals with lower-limb impairments, while also offering benefits such as augmented strength and reduced effort for unimpaired users [1]. The design and development of these devices require a multidisciplinary approach, with biomechanical modeling as a helpful conceptional framework. Biomechanical gait models help to better understand and analysis human locomotion, the interaction between the human body and assistive devices, and optimization of the design and control parameters to achieve optimal performance [2].

In the realm of biomechanical models, two main categories are commonly used: conceptual models and detailed models [3]. By conceptual models, we refer to computational models that provide an abstract representation of human movement patterns, e.g., the overall motion behavior of a whole limb, using basic mechanical elements and possibly minimalistic control circuitry. Detailed models include more intricate mathematical formulation to address different biomechanical details of human movement systems like musculoskeletal structure, actuation on muscle level and neural control. Both have been employed in the development of lower-limb assistive devices, offering valuable insights and contributing to advancements in the field [2, 4].

**Fig. 1:**
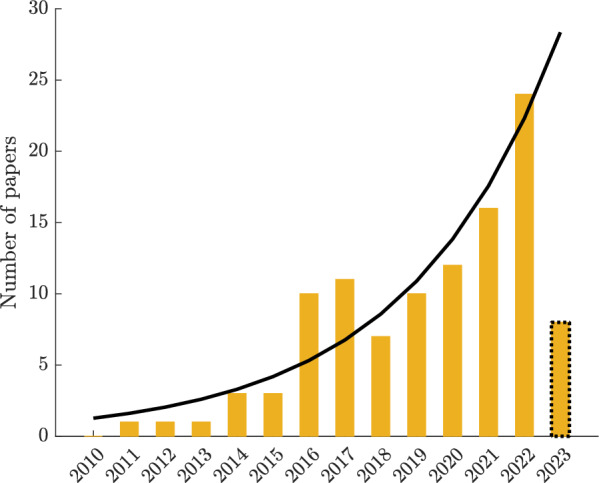
Number of papers per year focusing on applications of biomechanical models in lower limb exoskeleton development included in our review (see “Biomechanical models in developing assistive devices” section  for the search method). We covered only some part of 2023 (until end of June)

Several studies reviewed selected aspects of exoskeletons for gait assistance [1, 5] and biomechanical models of gait [6–9]. However, the potentials of biomechanical models have not yet been considered systematically enough in the development of assistive devices. For instance, usage of musculoskeletal modeling driven by electromyography (EMG) signals for designing personalized rehabilitation interventions and human–machine interfaces has been discussed in previous reviews, such as the work by Sartori et al. [10]. Some reviews briefly discuss the benefits of incorporating biomechanical models in predictive simulations for designing assistive devices [9, 11]. Grabke et al. [2] provided valuable insights into the role of musculoskeletal simulation in the design optimization of lower limb assistive devices, including exoskeletons and prostheses. Their work shed light on the significance of musculoskeletal modeling in this context. Our study aims to expand upon their research by incorporating a broader range of recent literature (see Fig. 1). Additionally, we aim to explore various other applications of biomechanical models in the development of assistive devices, beyond just design optimization. In recent years, the availability of efficient numerical methods and open-source software for predictive simulations based on complex models has significantly accelerated the development of assistive devices [11]. Figure 1 shows an exponential increase in the number of papers using biomechanical models in developing assistive devices. However, a comprehensive review specifically targeting the applications of biomechanical models in the development of lower limb exoskeletons is lacking, which is addressed in this article.

The purpose of this paper is twofold: first, to systematically review the existing literature on the applications of biomechanical models in the development of assistive devices; and second, to critically analyze these applications to identify gaps in the current research and propose potential directions for future work. Our review combines elements of both a systematic review, following predefined search strategies and selection criteria, and a critical review, where we synthesize findings to highlight areas that require further investigation [12]. By combining these approaches, this review aims to provide a comprehensive understanding of the field and guide future research efforts.

In the following, we begin by introducing different categories of biomechanical models in “Biomechanical gait models” section. “Biomechanical models in developing assistive devices” section details the specific methodologies used to systematically screen and select relevant literature for this review. Additionally, we discuss the applications of biomechanical modeling in the development of lower limb exoskeletons. Subsequently, in the final section, we will engage in a discussion of our findings and outline potential future directions in the field. By focusing on more recent research (shown in Fig. 1), this review intends to highlight the importance of biomechanical models for the advancement of lower limb exoskeletons and to support future research in this field.

## Biomechanical gait models

**Fig. 2:**
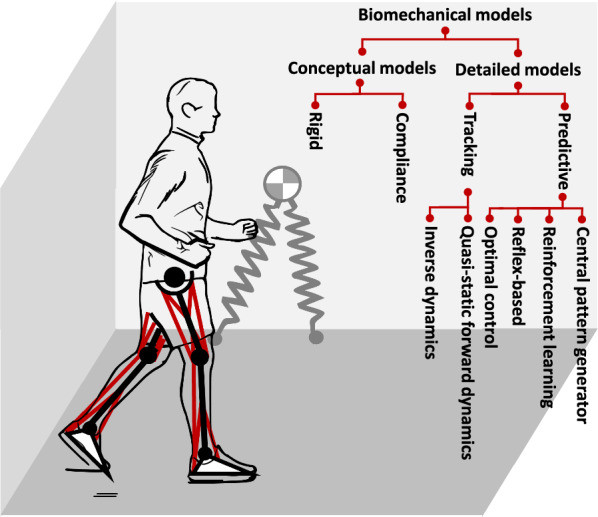
Overview of various biomechanical models for gait, classified into conceptual and detailed categories along with their subcategories. Conceptual models serve as abstract representations of complex mechanics of gait. Detailed models attempt to integrate the mechanical principles of biological systems with the neural control of these systems, providing a more complete picture of the underlying interactions between the nervous and musculoskeletal systems

Biomechanical models of gait encompass a range of models that aim to describe and understand human gait patterns and mechanics (see Fig. 2). The intricate nature of human gait arises from a complex, high-dimensional, and nonlinear interplay between the human body and its environment, posing challenges in the development of an accurate gait model [3]. To address the complexity of gait mechanics and motor control, conceptual models were developed to provide condensed representations of the underlying dynamics [13, 14]. These models offer a macroscopic view of locomotion tasks while capturing targeted key features of biological gaits. The main purpose of conceptual models is to capture the fundamental principles and relationships involved in human locomotion in a more accessible and manageable manner [3]. On the other hand, detailed models, such as musculoskeletal models, provide a more comprehensive representation of the human body and its underlying dynamic and motor control. These models incorporate skeletal segments, joints, muscles, tendons, and their associated properties and interactions [15]. With a more complex musculoskeletal system, these models enable a more detailed description of biomechanical factors influencing gait patterns and can be used to study various aspects of human locomotion, including muscle coordination, joint loading, and the effects of assistive devices. The list of conceptual and detailed models presented in this review is derived from the template anchor modeling paradigm [3] which is a well accepted categorization for understanding the biomechanics of human movement [16]. This section is intended to provide an introductory overview of the models that are relevant to the development of assistive devices.

### Conceptual models

In the context of human gait, a conceptual model offers the advantage of representing the fundamental characteristics of gait behavior in a parsimonious manner. This approach allows researchers to capture the underlying principles and mechanisms governing gait, without becoming entangled in intricate details pertaining to individual joints and muscles. By encoding body motion with respect to a minimal set of variables, a conceptual model effectively mitigates the challenge of high dimensionality, facilitating the study of the general system behavior [3]. Moreover, by presenting an intrinsic hypothesis concerning the high-level control strategy underlying a given task, a conceptual model facilitates a deeper understanding of the neural and biomechanical mechanisms that drive behavior. This concept known as “*Template* & *Anchor*” [3] is a methodology that leverages lessons learned from conceptual models to develop more detailed morphological and physiological models to investigate specific involvement of multiple legs, joint torques, muscle recruitment, and neural networks activation. Noteworthy examples include the revelation of the role of leg force feedback in posture control [17, 18], as well as the role of local positive muscle force feedback in governing leg stiffness [19]. Here, we divide conceptual models into two categories, namely compliant and rigid models.

#### Compliant conceptual models

Compliant conceptual models of gait [20], such as the spring-loaded inverted pendulum (SLIP) model [21], have proven to be valuable tools for understanding and studying the mechanics of gait. An illustrative example involves the utilization of a bipedal spring-mass model [13], which demonstrates how the compliant behavior of the stance leg during running can replicate the stance dynamics observed in walking, highlighting the ability of a shared mechanical system to exhibit distinct motor behaviors through the same mechanical system setup. Extensions of the SLIP model were presented by adding the trunk and damping in the leg [22, 23] to better represent human movement mechanics and motor control with template-based models. In [24], utilizing leg force feedback to adjust hip joint stiffness was introduced to stabilize the upper body around the supporting leg in running. This parsimonious control circuitry in the template models, which was presented as force-modulated compliance (FMCH) model of walking [17], could replicate the virtual pivot point (VPP) concept observed in human walking [25]. In [26], the effect of wobbling masses on impact force during running is investigated. These models also prove beneficial in identifying key characteristics of gait that may undergo modifications in diverse populations, including individuals with neurological or musculoskeletal impairments [27].

#### Rigid conceptual models

Rigid conceptual models mostly drive from the inverted pendulum model [28] which approximates the center of mass of the body as a point mass located above a pivot point and is subject to gravitational forces. While these models do not correctly represent the stereotypical double-hump ground reaction of human walking, it helps elucidate fundamental principles and relationships. For instance, the inverted pendulum model helps describe the mechanical work required to redirect the velocity of the center of mass during step-to-step transitions and metabolic determinants of the preferred step width in human walking [14, 29]. Also, this model is utilized to estimate metabolic cost of gait [30]. Notably, the inverted pendulum models have also contributed to understanding important principles governing gait stability. For instance, passive leg dynamics offer stabilization in the sagittal plane during walking, while active control is necessary for lateral stability [31]. These models have also been successfully used to generate walking pattern for bipedal robots [32].

### Detailed models

These models encompass a range of approaches, some of which primarily address musculoskeletal dynamics, while others additionally strive to integrate neural control. By doing so, they aim to offer a more comprehensive understanding of the intricate interactions between the nervous and musculoskeletal systems. Also, complex musculoskeletal models provide a detailed framework for exploring how the neuro-musculoskeletal system manifests the principles elucidated by conceptual models [4]. Accurate modeling of the musculoskeletal system is important in comprehending both pathological and healthy gait, as well as investigating the interplay between the musculoskeletal and neural systems [33]. Moreover, these models have potential in personalized clinical decision-making and the design of assistive technologies such as exoskeletons [11]. Simulation approaches using detailed models can be categorized into two main types: tracking simulation and predictive simulation.

#### Tracking simulation

Tracking simulations refer to a kind of simulation wherein the errors between simulated and experimental data are minimized or constrained. The objective of tracking simulations is to reproduce the observed behavior and to estimate muscle forces, activations, and contributions to joint motion.

- Inverse dynamics: Inverse dynamics calculate the generalized forces, such as net forces and torques, responsible for a specific motion. This approach utilizes experimental measurements of subject kinematics and external forces to estimate the acceleration of the body segments within the model by performing double differentiation of joint angles and positions. The estimated acceleration, combined with external forces like ground reaction forces, is then incorporated into the model’s equations of motion to solve for the internal joint forces and moments necessary to produce the observed motion. The process entails formulating an optimization problem to decompose the net joint moments into individual muscle forces at each time instant, commonly referred to as static optimization [34]. Static optimization assume that the musculoskeletal system is in static equilibrium at the desired pose and simplifies the problem by ignoring the subsequent dynamics of the movement. Therefore, this method assumes stiff/rigid tendons and does not account for activation dynamics.
- Quasi-static forward dynamics: To incorporate activation dynamics, a common approach is to utilize dynamic optimization techniques to identify a combination of muscle excitations that best reproduces experimental data [35]. Computed muscle control (CMC) is an alternative approach that involves solving a series of static optimization problems to determine the muscle excitations required to follow a given kinematic trajectory. CMC also incorporates a proportional-derivative (PD) controller to refine muscle excitations in order to reduce tracking errors between experimental and predicted joint angles. CMC has demonstrated successful applications in various musculoskeletal models and movement tasks, including walking, running, and jumping [36–38]. Notably, this method also permits the inclusion of additional inputs, such as EMG and ultrasound data, as control signal for muscles or to estimate model parameters, like for musculotendons as part of the inverse problem [39, 40].

#### Predictive simulation

Predictive simulations refer to a kind of simulation which do not necessarily require experimental movement data as input.

- Optimal control: Predictive simulations often assume that the central nervous system can be centrally controlled to optimize performance by minimizing suitable functions, such as the metabolic cost of transport. Within this framework, predictive simulations can be formulated as optimal control problems [11]. This approach involves solving for muscle controls in an open-loop manner, commonly referred to as trajectory optimization. However, it is important to note that these approaches do not provide explicit descriptions of gait control policies and are thus inadequate for capturing how the neuro-musculoskeletal system handles uncertainties, such as sensorimotor noise and external perturbations [11, 33].
- Reflex-based: This approach involves the derivation of control policies that govern the relationship between muscle controls and the state of the musculoskeletal system, thereby encompassing feedback control. These control policies are established based on the integration of principles originating from legged mechanics [4]. Approaches that aim to solve for gait control policies offer the ability to capture the robustness exhibited by the neuro-musculoskeletal system when confronted with various sources of noise and perturbations [41, 42].
- Reinforcement learning: Reinforcement learning (RL) emerges as an alternative approach to generate control strategies for forward dynamics simulations in an automated fashion. RL constitutes a machine learning technique employed to address decision-making problems by aiming to learn an optimal policy. This policy enables an agent to maximize its cumulative reward through interactions with the environment. In the context of learning human locomotion, the RL environment is represented by the musculoskeletal system and the physics-based simulation environment. The reward function employed in RL could addresses specific objectives such as achieving a target velocity with reduced muscle effort, among others [43]. Moreover, RL methods may incorporate learning from reference or imitation learning to ensure that the acquired optimal policy exhibits natural human-like behavior [44].
- Central pattern generator: Central pattern generator (CPG) utilizes oscillatory neural circuits to generate rhythmic and coordinated muscle activations, enabling tasks like walking, running, or other cyclic movements without continuous input from higher brain centers [45]. Also, feedback from somatic senses, such as joint angles and foot-ground contact signals, is then feedback to the neural system, which, in turn, regenerates the neural rhythm pattern based on this information. This theory posits that human locomotion arises from the cooperative interplay between neural rhythm and the dynamic rhythm of the body [46]. In [47] the idea of central pattern generator is combined by muscle synergy hypotheses to generate muscle excitations that can effectively reproduce both walking and running behaviors in a human musculoskeletal model.

## Biomechanical models in developing assistive devices

Biomechanical models can play a significant role in the development of assistive devices by providing valuable insights and tools for design, optimization, and evaluation. For the scope of this review, we focused on exoskeletons that work in conjunction with the biological joints, excluding prosthetics and full body exoskeletons that support complete limb function.

**Fig. 3:**
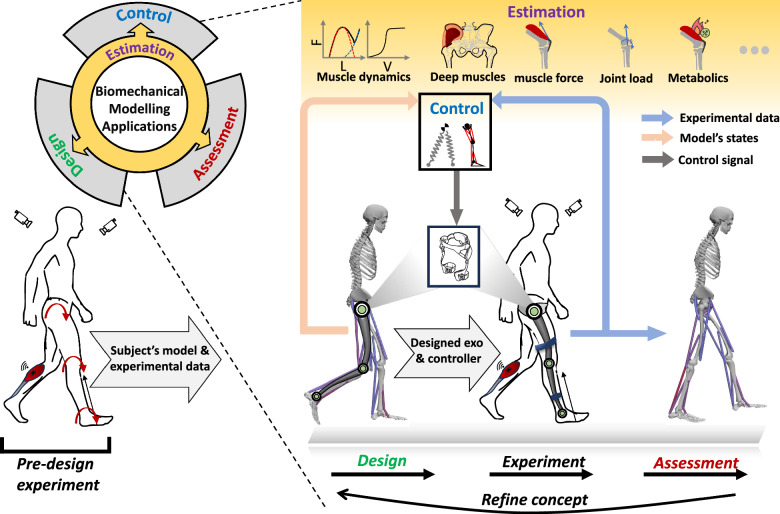
Applications of biomechanical gait models in the development of lower-limb exoskeletons for design, control, assessment and estimation. Estimated variables (e.g., muscle activation, muscle dynamics, muscle force, joint load, metabolic cost, etc.) can be used in other mentioned applications. By leveraging biomechanical models, these applications contribute to the design and refinement of assistive devices that enhance gait in both impaired and unimpaired individuals. Predesign state refers to experimental data collection for model development and tracking simulation in the design process. We used OpenSim software to generate visualization for the musculoskeletal model [48]

Most of the publications were found using the following Google Scholar query: (“*gait model” OR “neuromuscular model” OR “musculoskeletal model” OR SLIP OR “inverted pendulum” OR “hill-type”) AND (ortho OR exos OR “wearable robot” OR “assistive device” OR “rehabilitation robot” OR assistance) AND (gait OR locomotion OR lower limb)* among the records published since January 2010 up to the end of June 2023. Articles published prior to 2010 were excluded to ensure the review covered recent advancements and current trends in the field. This cutoff was chosen based on the rapid development of modeling techniques in recent years, which has significantly influenced the conceptual and detailed models we discuss (see Fig. 1). First, we screened the references based on their titles, followed by abstracts, and finally, the full-text to determine their relevance to our review’s scope. Subsequently, we thoroughly read and categorized the selected articles into four application areas: (1) Design, (2) Control, (3) Assessment, and (4) Estimation (see Fig. 3).

### Design

Given the high costs and time demands involved in human experiments, biomechanical modeling could serve as a valuable tool to design assistive devices and test ideas regarding assistance strategies. Also, during the design process and before prototyping an assistive device, it is crucial to predict the effects it may have on the users due to the close interaction between the human and the device.

In a recent study by Koelewijn et al. [49], virtual participants were used in a musculoskeletal simulation to replicate human gait adaptations and changes in energy expenditure observed during walking with an exoskeleton. This study demonstrated the potential of musculoskeletal simulation in accurately predicting the effects of assistance on gait and energy expenditure. Robertson et al. [50] utilized a predictive model of human neuromechanical adaptation to a passive elastic exoskeleton applied at the ankle joint to investigate the effects of exoskeleton stiffness on muscle-tendon mechanics and energetics. Similarly, Bianco et al. [51] conducted a study to examine the effects of ankle exoskeleton assistance on center of mass kinematics through predictive simulation, offering insights for designers aiming to enhance balance stabilization during walking. Another study by Han et al. [44] employed predictive simulation to assess the effects of an ankle exoskeleton on physical human-robot interaction (pHRI). In [52], the effects of different controller on the interaction force between human and exoskeleton are investigated. Predictive simulation is also used to study the performance of an assist-as-needed controller regarding the reductions in the metabolic cost and muscles’ activity [53]. In [54], a simulation study conducted to assess the effectiveness of impedance control applied to a lower limb exoskeleton in assisting disabled people during gait swing phase. In [55] the functionality of a passive load carrying exoskeleton tested in simulation environment. The simulation results show that the exoskeleton can reduce the foot pressure of the users, which validated through experiment. Some studies considered tracking simulation to predict the effects of an exoskeleton on metabolic cost and muscle activities [37, 56–58]. A tracking simulation of an unpowered ankle-foot exoskeleton shows the potential of the device to both reduce muscle forces and powers [59]. The simulation results in [60] demonstrate that a passive knee exoskeleton effectively reduces the exerted force on the extensor knee muscle and calf plantarflexion muscle during gait. These findings have been experimentally validated. Also, a tracking simulation was used in [61] to estimate the effects of a knee exoskeleton on knee contact force. This study shows a trade-off between interaction forces and physiological torque, which should be considered when developing controllers for the knee exoskeleton.

One of the crucial steps in designing assistive devices is determining which joints to assist, a decision influenced by the specific application and user requirements. For instance, several studies used tracking simulation to study the effects of ideal assistance in different application and on different users. In Dembia et al. [37], an optimization approach was utilized to determine the optimal assistance for an ideal actuator supporting lower limb joints. They found that devices providing assistance for hip flexion, knee flexion, and hip abduction have greater potential regarding metabolic benefits compared to the ankle plantar flexion device during load carrying [37]. In a similar study, Liu et al. [62] developed an ideal actuator that delivered assistance torque proportional to the human joint torque during load carrying. Results of this study show that hip extension assistance has more advantage regarding metabolic reduction, especially on slope, which is in contrast with the results from [37] that shows the advantage of assisting hip flexion in load carrying. This observation suggests that the optimal assistance could deviate from the biological joint torque. Uchida et al. [38] employed a similar approach as Dembia et al. [37] to investigate the effects of ideal assistance in each joint during running at different speeds. The results of their study suggested that for slow running, assisting more distal joints was advantageous, while for fast running, assisting more proximal joints was beneficial in terms of reducing metabolic costs. In [63] a walking assistance using optimized timed forces at the waist is investigated, inspiring from a conceptual model of human gait. Biomechanical models were also applied to design assistive device for pathological gaits. For example, musculoskeletal modeling of ideal assistance suggested that providing hip support to the elderly during walking could result in significantly greater metabolic benefits compared to ankle support [64]. Lim et al. [65, 66] employed a predictive simulation based on dynamic optimization to examine the potential advantages of hip exoskeletons in assisting gait with ankle pathology.

Moreover, biomechanical simulation can be used to guide exoskeleton structure and arrangement design. For instance, an estimation of muscle forces based on a tracking musculoskeletal simulation is employed to inform the design of cable paths in an exosuit [67]. In [68], a bionic muscle was designed by inspiration from Hill-type muscle model to assist the ankle and knee joints. Tracking simulation also employed in [69] to investigate the advantages of hybrid actuation in designing lower limb exoskeletons to reduce the metabolic cost of walking with heavy loads. Additionally, Rosenberg et al. [70] explored the potential impacts of powered and passive ankle-foot orthoses on muscle demand in children with cerebral palsy and crouched gait using a tracking musculoskeletal simulation. Nguyen et al. [71] utilized a fully predictive simulation based on reflex control to investigate the effects of unilateral and bilateral ankle assistance. Kim et al. [72] developed a mechanism for an exoskeleton specifically designed to assist running gait, leveraging the representation of running gait using a spring-loaded inverted pendulum model. Another design aspect related to the arrangement is single versus multijoint interaction. For instance, Bianco et al. [73] demonstrated through musculoskeletal simulation that coupling degrees of freedom can yield similar metabolic benefits as independently controlled multi-joint devices. Their findings suggest that coupling degrees of freedom can simplify control while still maintaining metabolic advantages comparable to multi-joint devices. Also, a predictive simulation [74] shows that ankle-knee multi-joint assistance is more effective in reducing the metabolism of human walking on slope compared to the single-joint or other combination of two-joint assistance. In [75] a fully predictive simulation is used to investigate the impact of single and multi-joint passive exoskeleton assistance on vertical jumping height.

Optimizing design parameters like mechanical elements properties and geometrical parameters of an assistive device allows for searching the best possible design that maximize the desired outcomes. Moreover, optimization enables designers to account for individual variability and user-specific customization of design. One approach is to try different combination of design parameters to find optimal design, which is time-consuming [76]. Another approach to tune design parameters of an assistive device is human-in-the-loop (HIL) optimization, which vary the design parameters systematically dering the experiment to find optimal parameters [77]. For instance, in [77], a HIL optimization is used to personalize an ankle-foot prosthetic stiffness in order to reduce metabolic cost of walking. Biomechanical models can also provide such a framework for the designer by considering the assistive device in the simulation of user movements and analyzing biomechanical data to find optimal design parameters that maximize the device’s effectiveness. Fang et al. [78] and Khamar et al. [79] proposed a general HIL optimization approach based on the MATLAB-OpenSim interface, which enables the optimization of geometry and material parameters for wearable robots. This framework was validated through the design of a knee exoskeleton for assisting flexion/extension movements [80]. In [81], a tracking musculoskeletal simulation was utilized to optimize the parameters of a serial elastic actuator. Similarly, Aftabi et al. [82] employed a tracking simulation study to investigate the effects of different hip exoskeleton spring stiffness on metabolic cost and fatigue during running gait. Ostraich et al. [83] investigated the effects of different knee exoskeleton stiffness on hopping height using predictive simulation. Guan et al. [84] investigated the effects of optimizing spring stiffness in a passive hip device on muscle activities for patients with spinal cord injury. In order to identify the most effective geometry for assistive devices, Marconi et al. [85] and Liu et al. [86] utilized musculoskeletal simulation to explore the effects of mass and mass distribution of the device. They showed the importance of device mass distribution on muscle activations. Another study employed musculoskeletal simulation to optimize the anchor point position of a cable-driven exosuit to reduce muscle activities [87]. Similarly, Guan et al. [84] investigated the effects of spring attachment point in a passive hip device on muscle activation for patients with spinal cord injury. The study conducted in [88] examined the impact of misalignment between the knee exoskeleton and the user’s biological joint on knee muscles through simulation. The findings indicate that such misalignment can lead to an increase in the force generated by the vastus lateralis muscle. Additionally, biomechanical models facilitate sensitivity analysis on the design and control parameters of assistive devices. By simulating the device’s performance under various conditions and manipulating its design or control parameters, designers can identify the parameters with the most significant impact on performance and make informed decisions regarding optimization. For instance, Hegarty et al. [89] conducted a simulation study to quantify the effects of assumptions about mechanical properties (e.g., rotational stiffness, damping, and equilibrium angle) of ankle-foot orthoses (AFOs) on the estimation of lower-limb muscle forces during stance in children with cerebral palsy. Their findings revealed that AFO stiffness had the more substantial effect on muscle force compare to the damping and equilibrium angle.

### Control

**Fig. 4:**
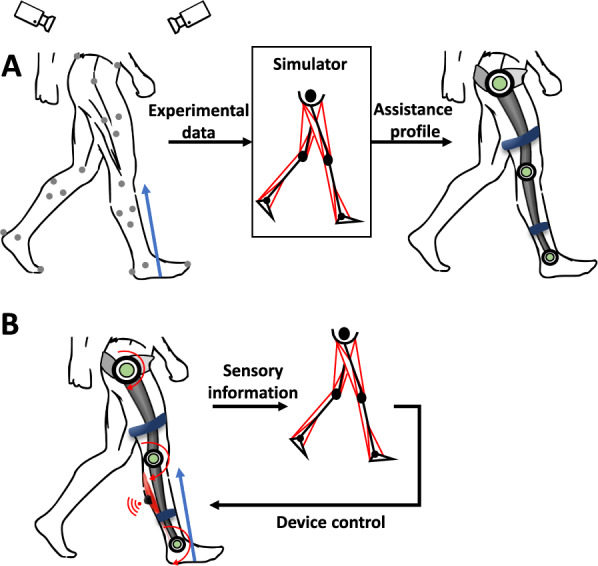
Using biomechanical models to design a controller for assistive devices. **A** Offline design of the desired trajectory. **B** Adaptive model-based control

The use of biomechanical models to control assistive devices holds great potential for improving the performance and usability of these devices. By incorporating biomechanical models, assistive devices could offer more natural assistance to users by considering their movement dynamics. Here, we review the uses of biomechanical models in desired trajectories design and in adaptive model-based control (see Fig. 4).

#### Offline trajectory designing

One of the approaches in designing controller for exoskeletons is to use a predefined assistance profile [90]. A state-of-the-art approach to design assistance profile is to parameterize a general profile and optimize these control parameters in a HIL optimization [91–94]. Biomechanical simulation can be used to simulate the human musculoskeletal system and the exoskeleton in order to tune the control parameters. Ratnakumar et al. [95] utilized a musculoskeletal model with a reflex-based controller [4] in a predictive simulation to optimize a parameterized hip assistance torque profiles. The same approach employed in [74] to optimize control parameters of single-joint or multi-joint assistance during slope walking. Afschrift et al. [96] used the same model to tune control parameters for an assistive device aimed at improving balance. Experimental results of this study show reduced center of mass (CoM) deviations in perturbed walking while using CoM velocity feedback in the controller compare to the same controller without CoM feedback. Lim et al. [65] used dynamic optimization simulation to optimize control parameters to reduce energy expenditures in pathological gait with a hip exoskeleton.

Biomechanical simulation can also be used to identify the optimal assistance pattern or trajectory that aligns with the desired objectives without parameterizing the profile. Dembia et al. [37], Uchida et al. [38], and Bianco et al. [73] employed detailed neuromuscular models to determine the optimal assistance trajectory for an ideal actuator, aiming to minimize metabolic cost while assuming the gait kinematics and kinetics remain unchanged with assistance. Franks et al. [97] employed the same approach to identify optimal assistance trajectories and conducted experimental tests with three subjects wearing the assistive device. The results of their study demonstrated that an optimized profile for a hip-knee-ankle device can reduce metabolic cost during walking up to $26\%$, showing the potential of this method. Although this approach achieved notable reductions in metabolic cost, it fell short of the results obtained through HIL optimization using the same device, highlighting the need for further improvement. Another study by Lee et al. [98] investigated the reduction in metabolic cost during running gait with an exosuit by comparing a simulation-optimized actuation profile based on Uchida et al. [38] with a profile derived from scaled-biological hip moment data. The findings revealed a higher reduction in metabolic cost when employing the simulation-optimized actuation profile, emphasizing its effectiveness in designing assistance profiles for assisting running gait. In another approach, instead of optimizing the assistance profile directly, some studies have considered the use of estimated muscle force to design the assistance profile. For instance, Wang et al. [99] developed an assistance force profile for an ankle exosuit based on simulated soleus muscle force. In this approach, a tracking simulation is utilized to estimate the force exerted by the soleus muscle. Subsequently, leveraging the simulated muscle dynamics, a scaled version of the force primarily generated by soleus muscle contraction is employed as the assistance profile.

#### Adaptive model-based control

In adaptive model-based control, a model is utilized within the control loop to generate assistance based on sensory feedback from the user. This section categorizes adaptive model-based control methods as follows:

- Reflex-based control: Here, controllers are derived from models of human locomotion that incorporate principles of legged mechanics. One prominent model is the gait model introduced by Geyer et al. [4], which is controlled by muscle reflexes and capable of producing different human gait patterns in a stable manner even in the presence of disterbance. An extended version of this model [100] has been successfully used to control leg prostheses, as demonstrated in Thatte et al. [101]. Researchers have also explored the applicability of this model’s controller version in exoskeletons. Wu et al. [102] conducted a preliminary evaluation of this controller on a lower-limb knee and hip robotic gait trainer, showing improvements in gait for subjects with incomplete spinal cord injuries. In Tamburella et al. [103], an ankle exoskeleton is controlled based on the reflexes of the soleus and tibialis anterior muscles to assist individuals with incomplete spinal cord injuries. The same controller is employed in Dzeladini et al. [104] to assist healthy subjects. Shafer et al. [105] designed an ankle exoskeleton controlled by positive force feedback to assist healthy individuals during walking gait, investigating the effects of varying gain and delay in the reflex loop on metabolic cost. The idea of positive force feedback combined with an adaptive fuzzy interface to personalized assistance from an ankle exoskeleton [106]. The reflex model introduced by Geyer et al. [4] was further extended by incorporating a center of mass velocity reflex to the ankle joint, enhancing its ability to replicate human ankle torque responses to perturbations [107]. This extension was later utilized to design a controller for an ankle exoskeleton to improve balance [96]. The concept of assisting balance using reflex-based control was also explored in Yin et al. [108] by incorporating a center of mass position reflex into a virtual neuromuscular controller to improve standing balance.
  Another group of reflex controllers is developed based on ground reaction force feedback, referred to as force modulated compliance (FMC) control [17]. In this approach, a reflex mechanism based on ground reaction force is employed to adjust the stiffness in the assistive device, drawing inspiration from the observed virtual pivot point concept in human gait and conceptual models of human locomotion [18, 25]. Zhao et al. [109] implemented this method on a gait training exoskeleton to provide assistance at the hip joint. Also, in [110], ground reaction force was used to modulate the stiffness in both the hip and knee joints of a gait training exoskeleton. Sharbafi et al. [111] employed FMC to drive a biarticular assistive device mounted in parallel to the hamstring muscle in a predictive simulation study, assisting both the hip and knee during the stance phase. This controller was extended to drive biarticular actuators parallel to both the hamstring and rectus femoris muscles in the design of BATEX (biarticular thigh exosuit) in a tracking simulation study [57]. The FMC controller was also utilized in a tracking simulation to control the assistance level in an assistive device acting on hip abduction [112].
- EMG-based control: In this approach, the EMG signal from the subject is used as the control input to a musculoskeletal model, which then provides real-time estimates of biological joint torques. A portion of these estimated joint torques is utilized to dynamically control the generated torques. Durandau et al. [113] employed this method to assist the knee and ankle joints of patients with post-stroke and incomplete spinal cord injuries using a multifunctional robotic exoskeleton in real time. The study utilized measured EMG signals from eight muscles to compute neural activation for 12 muscle-tendon units in a subject-specific musculoskeletal model, enabling the estimation of knee and ankle joint moments. Fleischer et al. [114] designed and controlled an exoskeleton for knee joint support based on estimated knee torque using EMG signals from six muscles acting on the knee joint and a musculoskeletal model of the knee joint. The same approach was implemented to drive an ankle exoskeleton in Durandau et al. [115], providing assistance to a healthy subject and resulting in reduced muscle activities. A similar experiment was conducted in [116], demonstrating the ability of the proposed framework to provide real-time information about joint work and underlying muscle mechanics. The experiment conducted in [115] was extended to include multiple subjects and tested under various walking conditions and transitions, without the need for redoing the calibration step [117]. The results of this study indicated that the proposed controller enabled individuals to voluntarily control a robotic exoskeleton across a continuous range of locomotion conditions while reducing muscle activation. In another study, Karavas et al. [118] developed a detailed subject-specific musculoskeletal model of the human knee to best match the user’s kinematic and dynamic behavior, enabling the estimation of knee joint moments. The estimated torque in [118] was then used to control the impedance of an exoskeleton. In [119], a musculoskeletal model of the knee joint is used to control a rehabilitation device based on EMG signals. The objective of the study was to develop an adaptive control strategy for knee rehabilitation that can provide assistance or resistance as needed during different phases of therapy. In [120], the authors utilized an EMG-driven musculoskeletal model to estimate the voluntary torque of the ankle joint. This estimated torque was then incorporated into an assist/resist-as-needed controller, resulting in a higher human contribution ratio. Furthermore, [121] demonstrated that utilizing an EMG-driven musculoskeletal model in an assist/resist-as-needed controller enables more natural and human-like human-robot cooperation compared to linear proportional EMG control.
- Artificial motor primitives-based control:
  Motor primitives in the context of locomotion are the fundamental building blocks or elemental patterns of movement that combine to form complex locomotion patterns [122]. In this approach, motor primitives are utilized to generate stimulation signals for a musculoskeletal model [123]. These signals are then transformed into muscle-tendon forces, which are further converted into joint torques. A portion of these computed torques is employed as assistance. The controller in this method is adaptable to the subject’s gait patterns, as it relies on the actual kinematics of the users to estimate muscle-tendon length. This approach has been successfully applied to control a hip flexion/extension exoskeleton [123–125].

### Assistance assessment

The current experimental methods for investigating the cause-and-effect relationship of assistive devices in human gait still face limitations due to the invasiveness of some measurement techniques and the complex interaction between different systems. The utilization of biomechanical modeling can provide valuable support and a deeper understanding of the effects of assistive devices on human locomotion, ultimately enhancing device design. These models target the simulation of device and user behavior and facilitate the quantitative assessment of device performance with respect to e.g. energy efficiency, injury risk, and comfort. For example, as high knee contact force during locomotion can lead to increased stress and knee damage, several studies employed musculoskeletal simulations to evaluate the effects of assistance on knee contact force [126]. In [127], a musculoskeletal simulation is used to study how the developed exoskeleton to assist stair climbing affects the knee contact force. Mclain et al. [126] demonstrated that a powered knee exoskeleton has the potential to reduce knee load by minimizing the required muscle work. In a study by Yamamoto et al. [128], musculoskeletal simulation was used to investigate the effect of changing the plantar flexion resistance of an ankle-foot orthosis on knee joint reaction and knee muscle forces. The peak knee joint reaction force in the early stance phase significantly increased in the strong plantar flexion resistance condition compared to no assistance, which may cause various knee problems such as knee pain and knee osteoarthritis.

Musculoskeletal simulation has also been utilized to explore the effects of assistance on muscle-tendon mechanics. In a study by Yap et al. [129], the impact of a knee brace on leg muscle forces was investigated through simulation and a significant difference was found in the muscle forces of the rectus femoris, gastrocnemius, soleus and tibialis anterior in assisted and not assisted conditions. Analyzing the effects of a passive ankle-foot orthosis on healthy gait using a musculoskeletal model shows that the device behaves more similarly to the soleus muscle and induces knee extension accelerations earlier in stance as the soleus typically exhibit [130]. In another study by Choi et al. [131], musculoskeletal simulation was used to analyze the effects of an ankle-foot orthosis with dorsiflexion resistance on Achilles tendon function during walking in healthy subjects. Musculoskeletal modeling also demonstrated that an ankle-foot orthosis can alter gastrocnemius operating length in post-stroke hemiplegic gait [132]. Musculoskeletal simulation was also utilized to analyze the effects of ankle exoskeletons in a hopping task, as demonstrated by Farris et al. [133, 134]. Furthermore, Jackson et al. utilized an EMG-driven musculoskeletal simulation in their study to explore how modifications in exoskeleton assistance influence plantarflexor muscle-tendon mechanics, thereby affecting metabolic cost [135]. In [136] a simulation study is employed to investigate how connecting the legs with a spring improves human running economy, as observed in experimental studies. Results of this study show that most of the savings occurred during stance phase and in muscles actuating more proximal muscles. In another study by Mo et al. [137], musculoskeletal simulation was employed to estimate the effects of an exoskeleton on the metabolic cost of post-stroke gait.

### Estimation

Biomechanical models could also be employed for estimating variables that are challenging to measure directly or are inherently unmeasurable in the context of assistive devices. These models offer valuable insights into various factors such as muscle forces, joint loads, joint moments, contact forces, and metabolic costs. This information can be utilized in the design, control or evaluation process of assistive devices. For instance, muscle models are employed to translate muscle activation into muscle force and joint torques [99, 117, 138, 139]. By incorporating EMG data, neuromusculoskeletal models have demonstrated robustness in estimating knee joint moments over an extended period in a multi-day experiment [140]. In another study [141], a combination of neuromusculoskeletal models with EMG signals and data-driven predictions of ground reaction forces and center of pressure was utilized to enhance joint torque estimation. Musculoskeletal simulations have been employed in [142] to compute muscle force and state estimates. These predictions can approximate the human cost function for optimization. For example, by estimating the metabolic cost using a musculoskeletal model, control parameters of a hip exoskeleton were optimized in the HIL optimization experiment [143]. Further applications of musculoskeletal modeling, e.g., in investigating joint loading, were summarized in a systematic review by Holder et al. [144].

## Discussion and future perspectives

Biomechanical gait models, ranging from conceptual models to more detailed models, hold significant potential in the advancement of assistive devices like lower limb exoskeletons. The utilization of biomechanical models enables researchers and engineers to gain deeper insights into the dynamics and control architecture of human movement, as well as the intricate interactions among muscles, bones, and tendons generating locomotion. This knowledge serves as a valuable foundation for designing novel or improved assistive devices that are safe, effective, and comfortable for users [103, 108].

Our review showed that biomechanical models have various applications in the design and development of exoskeletons: (1) Exo Design: Biomechanical models could guide exoskeleton design by identifying optimal joints for assistance, informing structural arrangements, optimizing mechanical properties and geometries, and allowing pre-prototyping evaluation through predictive capabilities. (2) Control: Biomechanical models could help to find the best assistance profiles for different applications and users using tracking or predictive simulations. They could optimize control parameters for specific control rules, and facilitating effective human–machine interaction through the incorporation of biomechanical models within the human-device loop [145]. (3) Assessment: Neuromuscular models derived from biomechanical modeling can be leveraged to evaluate the effects of assistive devices and establish cause-and-effect relationships between the device and the user. These models provide valuable information regarding the device’s effectiveness, efficiency, and potential areas that may require improvement. (4) State Estimation: Biomechanical models serve as a platform for estimating unmeasurable or hard-to-measure variables.

In the following, we discuss relevant areas which appear little researched yet as well as future perspectives on using biomechanical models in the exoskeleton field.

### Validation

Simulation models are simplifications of real-world systems and behaviors. The validation of a simulation model provides a high level of confidence in its suitability for a specific intended purpose [146]. This encompasses both qualitative and quantitative behavior. Validation ensures the reliability and accuracy of the simulation-based predictions. A few studies that have validated simulation results have demonstrated the significant potential of designing assistive devices and controllers using biomechanical models. For instance, a simulation-optimized assistance profile for assisting the hip joint during running yielded greater metabolic benefits compared to a generic assistance profile [98]. Similarly, the application of a simulation-optimized assistance profile on a hip-knee-ankle exoskeleton during walking resulted in a reduction in metabolic cost [97]. These validation studies highlight the potential of biomechanical simulation in predicting the response to assistance, as demonstrated in the use of an ankle-foot orthosis in a healthy subject [147]. Although some experimental studies have not directly compared their results with simulation studies, they have demonstrated satisfactory agreement. For example, an experimental study investigating multi-joint assistance revealed that assisting the hip, knee, and ankle simultaneously could lead to the largest metabolic reductions, while the combination of hip-ankle, knee-ankle, and hip-knee assistance resulted in progressively lesser metabolic benefits [148]. These experimental findings align with the results obtained from simulation studies [73]. Integrating a musculoskeletal model within the control loop has also shown promising results, including reduced muscle activity and the ability to adapt to various conditions [117].

**Fig. 5:**
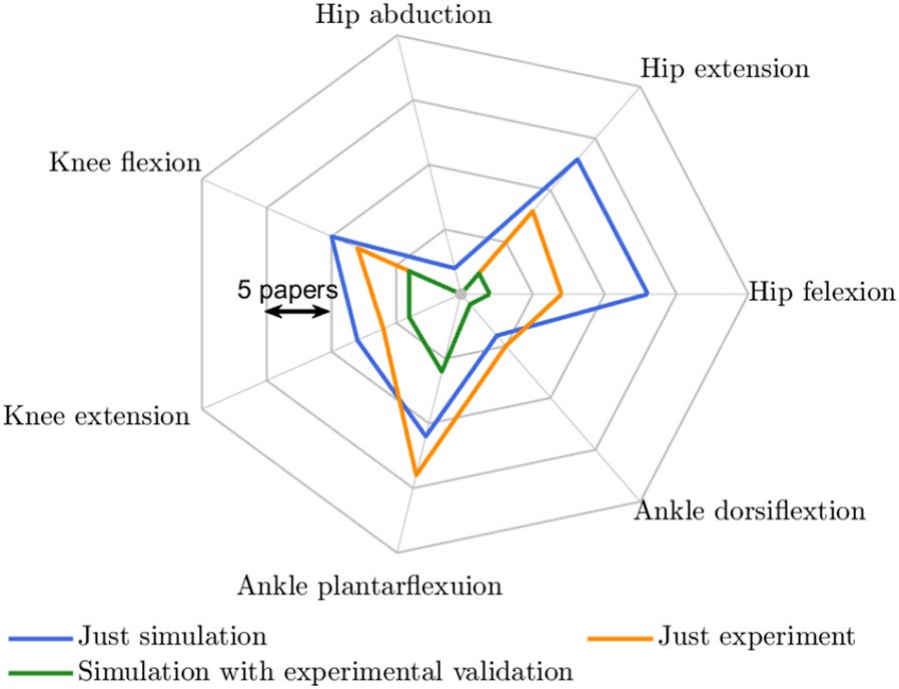
The number of reviewed papers that designed exoskeletons and/or controllers based on biomechanical models. Just simulation means the designed exoskeletons and controllers are tested in simulation without experimental validation. The green curve shows papers that designed an exoskeleton and/or controller in simulation and validated it in an experiment. The orange curve shows papers that used biomechanical models in a control loop without testing it in a simulation environment. The total curve shows the total number of papers which considered designing a controller based on biomechanical models for a specific degree of freedom. Each level in this graph represents five papers

While the optimization and control of assistive devices using musculoskeletal models hold theoretical promise, there is a lack of rigorous experimental validation for the design and control of assistive devices. For instance, the advantages of assisting hip abduction in load-carrying tasks shown in simulations [37, 112], have never been validated with human experiments, as seen in Fig. 5. This figure indicates a discrepancy between the number of studies focusing solely on simulation versus those incorporating experimental validation. A significant proportion of studies on devices assisting proximal joints (e.g., hip) rely solely on simulation (blue curve). Studies that solely consider experiments often involve the integration of biomechanical models within the control loop. The number of experimental studies is dominant in more distal joints, such as the ankle joint. This observation might be attributed to the relative ease of using biological signals like EMG for more distal joints compared to proximal joints [117, 149].

To improve gait assistance with the help of musculoskeletal modeling, further validation research is required. The required validation studies depend on the type of assistive device, the target user population, and the intended use case. Nevertheless, functionality validation by comparing the experimental outcomes with the simulation predictions (e.g. using emulators [93]) is advantageous. Also, comparing the simulation results with existing experimental results from existing state-of-the-art devices can be considered while validating simulation-based designed assistive device or controllers. For instance, in [150], the simulation result of finding optimal assistance patterns is validated based on the existing state-of-the-art human-in-the-loop optimization results [91]. Further, performing sensitivity analysis on the simulation model and parameters enhances result reliability and design validity. The use of different simulation tools and models of varying complexity also increases confidence in the consistency of the design.

### Improving models

Improving the fidelity of models is vital for the development of more practical assistive devices. Between the conceptual and detailed neuro-musculoskeletal models, the second group is utilized more for the design and control of assistive devices. These models consist of two main components: the musculoskeletal design and the neural control. The inclusion of personalization in the musculoskeletal modeling could enhance the model functionality in assistance enhancement and facilitate understanding of inter-subject variations in gait patterns [151]. Strategies such as extracting bony geometries and joint centers from magnetic resonance imaging scans [152], personalizing musculotendon parameters based on electromyography (EMG) or ultrasound data [39, 40], and customizing the location of muscle attachments [153] could improve a model’s accuracy. Another approach is to involve more biomechanical details of the body in the models. For instance, modeling the foot as a two-segment instead of a single-segment system has a negligible effect on knee torque estimation in inverse simulations while significantly impacting knee torque in predictive simulations [11]. The inclusion of more accurate dynamical models of the muscles can also improve the accuracy of simulation results [154]. Furthermore, integrating the viscoelastic behavior of soft biological tissue into digital human models holds promise for improving the design of wearable assistive devices, thereby increasing their efficiency and efficacy [155].

The next level which could make the neuromuscular models closer to the biological body is the inclusion of neural control. One common approach to modeling neural control and resolving the muscle redundancy problem is to optimize a performance criterion that represents the high-level goal of the motor task. Personalizing this optimization criterion can lead to improved simulation results. A potential solution for personalizing the optimization objective involves determining a weighted cost function from a family of possible cost functions, representing an inverse optimal control problem that can be addressed using a bilevel optimization approach [156]. In this approach, the lower level involves optimizing the targeted movement by minimizing the cost function subject to the dynamic equations of the musculoskeletal model. The upper level adjusts the weights for each cost function to minimize the discrepancy between the lower-level solution and experimental data [156–159]. Optimization-based neural control offers mathematical solutions that optimize performance according to predefined criteria without providing acceptable insights into the underlying neural control. However, when studying neurological disorders affecting gait performance, it is imperative to investigate the neural control structures involved in generating muscle activations [11]. Despite considerable efforts to uncover the underlying neural circuits and pathways associated with movement control [4, 41, 160], the complexity of developed control models still falls short of that observed in human motor control. In this context, translating findings from conceptual models into neural control [18, 161, 162], exploring novel ideas about neural control through perturbation studies [42, 107, 163], or developing mathematical models based on clinical definitions of neurological symptoms [11, 164] can improve simulation models’ prediction ability. Moreover, based on a simulation study, it is proposed that the integration of optimization and reflex excitation leads to improved predictions of experimentally optimized assistive torque profiles using human-in-the-loop optimization [150]. Additionally, considering uncertainty during gait simulation can make the models more realistic. For instance, the co-contraction of antagonistic muscle pairs, often observed in human movement and deemed inefficient in deterministic simulations, appears to minimize effort in systems with uncertainty [165].

When considering simulations of human movement augmented by assistive devices, incorporating a more precise model of the device can substantially enhance the predictive capabilities of the models. Research by Nguyen et al. [166] demonstrates that the electrical dynamics of the motor could critically influence simulation outcomes. Additionally, incorporating the dynamics of physical interactions, such as the viscoelastic properties of soft biological tissues, into biomechanical gait models is essential for accurately predicting exoskeleton performance. Neglecting these interaction dynamics can significantly degrade exoskeleton efficacy estimates and interpretations, as demonstrated by Yandell et al. [167]. Therefore, future research should prioritize integrating these dynamics into biomechanical models to minimize the sim-to-real gap and improve the design and control of assistive devices [155, 167]. Calibrating contact models can lead to a more accurate estimation of interaction forces between humans and exoskeletons, which is essential for assessing user comfort and the effectiveness of the interaction [168]. Additionally, it is worth noting that perfect alignment between the exoskeleton model and human joint axes is assumed in exoskeleton simulations. However, in practical exoskeleton usage, misalignment between human and exoskeleton joints is often observed, resulting in unrealistic simulation outcomes [169].

In summary, further research on modeling to be used for movement assistance applications could be done in different directions: (1) More detailed modeling: involvement of more intricate musculoskeletal simulations, (2) neural control: exploring new models to approach human neural control, (3) personalization: adaptation of the model parameters based on individual bodies and motor control, (4) cost function identification: exploring new approaches (e.g., multi-objective optimization or inverse optimal control) to identify biological cost function to optimize the models, (5) Stochastic modeling: Including uncertainty and noise in the models, (6) template modeling exploration: Extension of conceptual models and their combination with detailed models. These different research lines could help improve understanding of human motor control, realize more realistic predictions of human-exo interaction, and yield more synergistic cooperation of man and machine.

### Model-based design and control of assistive devices for pathological gait

**Fig. 6:**
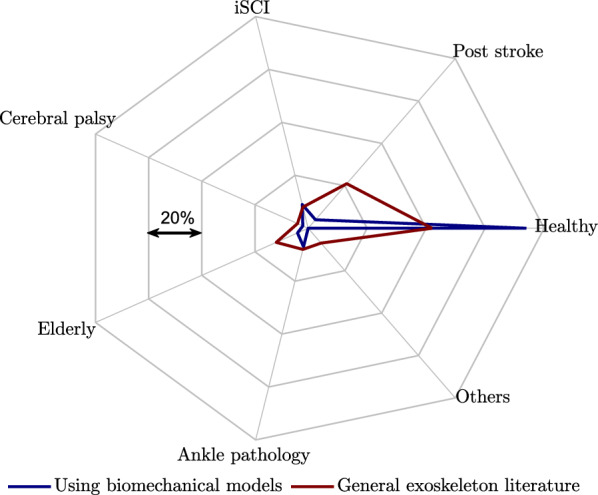
Distribution of user groups in lower limb exoskeleton research: biomechanical model-based design reviewed in this article (blue) vs. general exoskeleton literature review from [172, 173] (red). *“Others”* include multiple sclerosis, poliomyelitis, spinocerebellar degeneration patients and research papers that are not specifically categorized under the aforementioned user groups

One of the primary motivations behind developing exoskeletons was to assist impaired individuals and enhance their mobility, aiming to improve their quality of life [170]. A systematic study assessing the use of exoskeletons in individuals with multiple sclerosis demonstrated that exoskeletons could preserve gait speed, significantly enhance functional mobility, and mitigate perceived fatigue [170]. Similarly, powered lower limb exoskeletons have been investigated as a novel form of robotic therapy to target motor impairments in individuals with cerebral palsy and enhance their gait training [171]. Despite the growing body of evidence supporting the effectiveness of model-based designed assistive devices and controllers, our review shows a prevalent focus on studies involving healthy subjects, as shown in Fig. 6. This figure shows that using biomechanical models to inform design of lower limb exoskeletons for pathological gait have received less attention compared to the whole category of lower limb exoskeletons to assist pathological gait (the user groups identified through scanning two review paper on lower limb exoskeletons [172, 173]). Simulation studies have demonstrated promising results in modeling pathological gait [27, 33, 174]. Investigations on the modeling of pathological gaits and model-based design and control of assistive devices could highly enhance the quality of assisting patient locomotion.

Moreover, studies that do consider model-based design for pathological gait sometimes rely on validation through testing on healthy subjects [175]. However, experimental findings indicate that improving gait in healthy individuals using designed devices or controllers does not guarantee the same effectiveness in assisting pathological gait. For instance, increasing ankle push-off work with a powered prosthesis has been shown to reduce metabolic rate in non-amputees [176], but this does not necessarily translate to metabolic rate reduction in transtibial amputees [177].

### Design objectives of assistive devices

Lower-limb assistive devices serve the purpose of providing support, compensating for impairments, or assisting individuals in various tasks and activities. This study indicates that a majority ($69\%$) of the papers that utilize biomechanical models to design and control lower-limb assistive devices prioritize the objective of reducing metabolic cost and muscle effort. However, a comprehensive review study on the performance indicators of lower limb exoskeletons indicates a predominant focus on improving kinetics, kinematics, spatiotemporal parameters, and speed as the design objectives for general lower-limb exoskeletons, with only $16\%$ of papers considering the reduction of metabolic cost and muscle effort as a primary goal [173]. One possible explanation for this observation is that most model-based designs and controllers are developed and tested on healthy users (refer to “Model-based design and control of assistive devices for pathological gait” section). For this particular user group, the prioritization of reducing metabolic cost and muscle effort may be more relevant. However, different design objectives may take precedence when addressing pathological gait. Predictive simulation holds promise in uncovering the physiological factors contributing to altered gait, thereby aiding in the design of assistive devices for target groups with movement difficulties. Moreover, it allows for the investigation of the effects of assistive devices on gait improvement beyond the scope of reducing metabolic cost and muscle effort, thus incorporates other important design objectives [44, 174, 178].

**Fig. 7:**
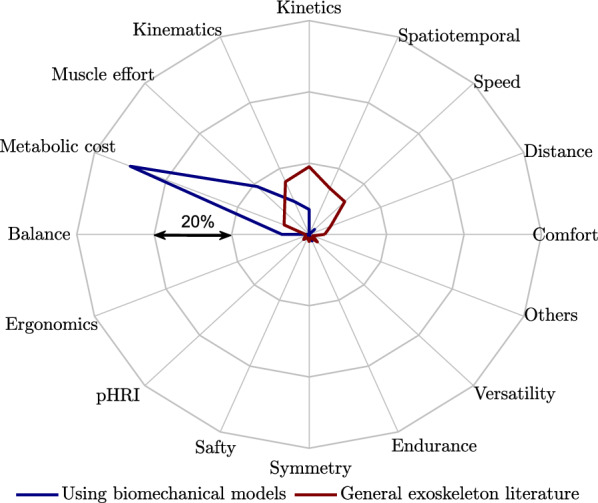
Distribution of design criteria in lower limb exoskeleton research. Biomechanical model-based design reviewed in this article (blue) vs. general exoskeleton literature review from [172, 173] (red). *“Others”* include cognitive effort, dependability and coordination

To improve user acceptance of assistive systems, considering subjective criteria is required, which received less attention in exoskeleton studies (see Fig. 7). In [179], user demands are considered in lower limb prosthetic design to improve user acceptance, which is transferable to the exoskeletons and can be considered in simulation studies. The parallel development of biomechanical models and the consideration of individual differences in body perception and psychological states can provide valuable insights into the interactions between the human body and robotic devices. This can lead to an improved realization of the seamless integration of the assistive device into the human body schema.

### Open-source data, model and software

Open-source data, models, and software have emerged as valuable tools for accelerating the development of assistive devices, facilitating the design process, and fostering collaborative efforts. An exemplary illustration of such open-source software is OpenSim, a freely available software enabling users to construct musculoskeletal models and conduct dynamic simulations encompassing a diverse range of movements [180]. By leveraging open-source models, researchers can circumvent the time-consuming and costly process of developing models from scratch. Furthermore, the presence of extensive online communities facilitates collaboration, knowledge sharing, and troubleshooting. In addition to enhancing collaboration and reducing costs, the utilization of open-source data, models, and software contributes to transparency and reproducibility within research endeavors.

The availability of open-source models and software packages has witnessed significant growth in recent years, exhibiting great potential for advancing the design and control of assistive devices [48, 181–184]. However, the scarcity of accessible datasets for developing and testing exoskeletons poses a challenge for researchers and developers seeking to create and enhance exoskeleton designs. This limitation hinders researchers’ ability to reproduce and build upon previous work. Notably, specific universities and research institutions have initiated the generation and sharing of their datasets, thereby making significant contributions to the progress of exoskeleton technology [185]. Additionally, some of these institutions are openly sharing their computer-aided design (CAD) and hardware specifications, facilitating experiment replication and fostering improvements in exoskeleton models through simulation [186].

Another promising approach is to use biomechanical models to generate datasets. For example, musculoskeletal models can produce synthetic data for data-driven methods, particularly in wearable technology. This synthetic data provides a valuable resource for training machine learning algorithms, allowing them to learn patterns and relationships within human movement without the need for extensive real-world data collection [187]. Additionally, musculoskeletal simulations can create highly controlled and varied scenarios-such as specific joint movements-that are challenging to replicate in real-life experiments.

### Role of assistive devices in the biomechanical models development

This paper highlights the immense potential of biomechanical models, ranging from conceptual models to detailed models, in advancing the development of exoskeletons. However, the utilization of assistive devices such as exoskeletons to enhance the predictive capabilities and accuracy of biomechanical models has received limited attention thus far. To improve biomechanical models, it is crucial to gain a deeper understanding of the characteristics and behavior of the human body. Perturbation study can serve as a valuable tool in this regard. Exoskeletons can be employed as perturbation devices to reshape human locomotion and investigate the resulting responses. For instance, in [188], a robotic exoskeleton was employed to shift people’s energetically optimal step frequency to frequencies higher and lower than normally preferred, with the aim of exploring whether individuals could optimize their energetic costs in real-time. Also, the same device is used in [189] to study how humans initiate energy optimization and converge on their optimal gaits. Findings indicate that, following a perturbation from their preferred gait, most participants tend to exploit their original gait, which is suboptimal now, rather than exploring the new energetic landscape. However, once providing them with the experience of lower-cost gaits, the nervous system can learn to predict this new optimal gait and rapidly return to it if perturbed away. In the works of Ahn et al. [190] and Baye et al. [191], exoskeletons were used to apply periodic mechanical perturbations during walking. These studies can advance our understanding of the control architecture governing human locomotion by exploring how participants adapt themselves to this perturbation. Furthermore, exoskeletons have been employed to identify subject-specific muscle parameters within a model, as demonstrated in [192, 193].

Using exoskeletons as perturbation tools, researchers can gain valuable insights into the characteristics and behavior of the human body, refine biomechanical models, and ultimately drive the development of exoskeleton technology.

## Data Availability

Not applicable.
